# Integrated treatment using intraperitoneal radioimmunotherapy and positron emission tomography-guided surgery with ^64^Cu-labeled cetuximab to treat early- and late-phase peritoneal dissemination in human gastrointestinal cancer xenografts

**DOI:** 10.18632/oncotarget.25649

**Published:** 2018-06-22

**Authors:** Yukie Yoshii, Mitsuyoshi Yoshimoto, Hiroki Matsumoto, Hideaki Tashima, Yuma Iwao, Hiroyuki Takuwa, Eiji Yoshida, Hidekatsu Wakizaka, Taiga Yamaya, Ming-Rong Zhang, Aya Sugyo, Sayaka Hanadate, Atsushi B. Tsuji, Tatsuya Higashi

**Affiliations:** ^1^ National Institute of Radiological Sciences, National Institutes for Quantum and Radiological Science and Technology, Chiba, Japan; ^2^ Division of Functional Imaging, National Cancer Center Hospital East, Chiba, Japan; ^3^ Research Center, Nihon Medi-Physics Co., Ltd., Chiba, Japan

**Keywords:** integrated ^64^Cu therapy, PET-guided surgery, OpenPET, ^64^Cu-intraperitoneal radioimmunotherapy, ^64^Cu-labeled cetuximab

## Abstract

Peritoneal dissemination is a common cause of death from gastrointestinal cancers and is difficult to treat using current therapeutic options, particularly late-phase disease. Here, we investigated the feasibility of integrated therapy using ^64^Cu-intraperitoneal radioimmunotherapy (ipRIT), alone or in combination with positron emission tomography (PET)-guided surgery using a theranostic agent (^64^Cu-labeled anti-epidermal growth factor receptor antibody cetuximab) to treat early- and late-phase peritoneal dissemination in mouse models. In this study, we utilized the OpenPET system, which has open space for conducting surgery while monitoring objects at high resolution in real time, as a novel approach to make PET-guided surgery feasible. ^64^Cu-ipRIT with cetuximab inhibited tumor growth and prolonged survival with little toxicity in mice with early-phase peritoneal dissemination of small lesions. For late-phase peritoneal dissemination, a combination of ^64^Cu-ipRIT for down-staging and subsequent OpenPET-guided surgery for resecting large tumor masses effectively prolonged survival. OpenPET clearly detected tumors (≥3 mm in size) behind other organs in the peritoneal cavity and was useful for confirming the presence or absence of residual tumors during an operation. These findings suggest that integrated ^64^Cu therapy can serve as a novel treatment strategy for peritoneal dissemination.

## INTRODUCTION

Peritoneal dissemination is the most common cause of death in gastrointestinal cancers, and conventional therapy provides limited benefits [1]. While no standard treatments are available for peritoneal dissemination, combining chemotherapy with cytoreductive surgery has been performed in clinical practice to treat this condition. However, traditional chemotherapy does not effectively diminish tumor lesions in the peritoneal cavity [2]. In addition, it is difficult to detect and remove tumors that are located deeply in the peritoneal cavity by cytoreductive surgery. Moreover, tumor masses in the peritoneum easily change their positions during an operation. These difficulties may translate to small effects on improving survival, particularly for late-phase peritoneal dissemination [3]. Therefore, a need exists for developing more effective drugs and improving surgical procedures to treat peritoneal dissemination.

Intraperitoneal radioimmunotherapy (ipRIT) performed with a tumor-specific binding antibody labeled with a therapeutic radioisotope, such as the β^−^ emitters ^131^I and ^177^Lu or the alpha emitter ^225^Ac, can potentially be used to treat small peritoneal metastases [4–6]. Previous reports have shown that intraperitoneal (ip) injection of radiolabeled antibodies resulted in high and rapid accumulation in ip tumors compared with that observed after intravenous (iv) injection and that ipRIT using an ^131^I-, ^177^Lu, or ^225^Ac-labeled antibody effectively prolonged survival in mouse models with small lesions of peritoneal dissemination [4–6]. However, ipRIT showed limited effectiveness against large peritoneally disseminated tumor masses, since the accumulation of radiolabeled antibodies decreased with increasing tumor volumes due to the low permeability of the antibody into tissues [4]. In addition, the production and availability of these radioisotopes remain key challenges. Especially, ^131^I and ^177^Lu is produced for medical use by a nuclear reactor, but sustainable use of a nuclear reactor is a critical issue for the future clinical practice [7, 8]. ^225^Ac is produced for medical use by radiochemical extraction from ^229^Th, and ^225^Ac production with a (p, 2n) reaction from ^226^Ra in a cyclotron has also been studied, although the availability of ^225^Ac is still limited [9]. Therefore, development of alternative radioisotopes for ipRIT is needed to increase utility of this therapy.

^64^Cu is a useful and practical theranostic radionuclide [10–12]. That is, ^64^Cu can be used for both positron emission tomography (PET) imaging and internal radiotherapy because it shows β^+^ decay (0.653 MeV, 17.4%), β^−^ decay (0.574 MeV, 40%), and electron capture (42.6%). The photons generated from electron–positron annihilation can be detected by PET, and the β^−^ particles and Auger electrons emitted from this nuclide can damage tumor cells [10, 11, 13]. We have shown that high-linear energy transfer Auger electrons emitted from ^64^Cu cause heavy damages to DNA in cancer cells [14]. Clinical PET studies using ^64^Cu-labeled agents, such as ^64^Cu-diacetyl-bis (*N*^4^-methylthiosemicarbazone) (^64^Cu-ATSM) [15] and ^64^Cu-labeled trastuzumab [16], have shown the utility of ^64^Cu for imaging in humans. Data from many preclinical studies have also demonstrated the therapeutic effectiveness of ^64^Cu-labeled agents, such as ^64^Cu-ATSM [15, 17], ^64^Cu-labeled Arg-Gly-Asp peptide [18], and ^64^Cu-labeled antibodies [19, 20]. Recently, a first-in-human study of radionuclide therapy with ^64^CuCl_2_ was conducted in Europe, and it was reported that the patient showed a remarkable reduction of tumor volume without side effects [21]. These lines of evidence support the applicability of ^64^Cu in clinical use for therapy. In addition, ^64^Cu can be easily produced using a biomedical cyclotron from ^64^Ni, and large-scale production for therapeutic usage is possible [19, 22]. Indeed, many production sites for ^64^Cu exist throughout the world [23]. Based on these unique features, we were interested in studying whether this nuclide could be used for ipRIT and PET imaging of peritoneal dissemination.

Recently, we developed the world's first open-type PET system, referred to as “OpenPET” [24, 25]. In this system, the detectors are arranged for sufficient open space to perform surgical procedures; in addition, the system can image and track objects with high-resolution PET in real time. For real-time imaging, this system performs high-speed reconstruction, which enables continual image updating in cycles of <1 s, while accumulating data. These unique features of this system enabled us to perform PET-guided surgery.

**Figure 1:**
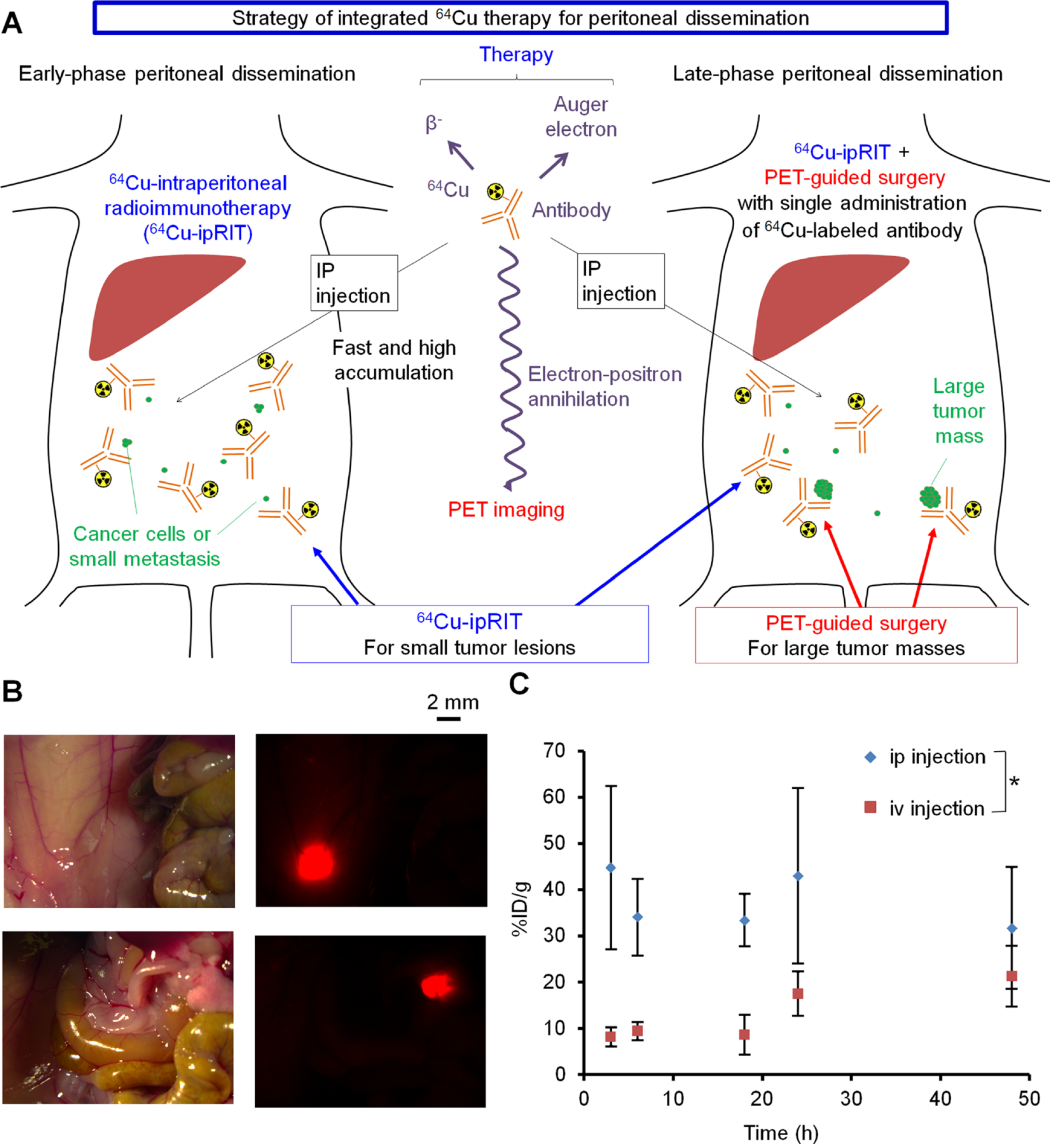
Strategy used for integrated ^64^Cu therapy (**A**) Schematic representation of the integrated ^64^Cu-therapy approach used for treating early- and late-phase peritoneal dissemination. (**B**) Small lesions in the early-phase peritoneal-dissemination mouse model with HCT116-RFP cells, detected with a stereoscopic fluorescence microscope. This model involved small tumors with diameters of approximately 2 mm or less. (**C**) Accumulation of ip- or iv-injected ^64^Cu-PCTA-cetuximab over time in small tumors in the early-phase peritoneal-dissemination mouse model with HCT116-RFP cells. Values are shown as the mean ± SD; *n* = 4 for each time point. ^*^P < 0.05 between the ip- and iv-injection results (2-way ANOVA).

By combining the advantages of ^64^Cu and OpenPET, we attempted to develop an integrated treatment using ^64^Cu-ipRIT or combining it with PET-guided surgery as a novel approach to treat early- and late-phase peritoneal dissemination in mouse models (referred to here as integrated ^64^Cu therapy) (Figure 1A). In this study, we used a ^64^Cu-labeled anti-epidermal growth factor receptor (EGFR) antibody cetuximab to obtain proof of concept for the integrated ^64^Cu therapy, since cetuximab has a high binding affinity for EGFR (which is overexpressed in a wide variety of cancers) and is commonly used to treat these cancers in clinical practice [26]. Additionally, ^64^Cu-labeled cetuximab has been well studied in many preclinical studies [20, 27–30] and has been useful for imaging [27–30] and internal radiotherapy via intravenous injection [20] in xenografted mouse models. For integrated ^64^Cu therapy, ^64^Cu-ipRIT was used against early-phase peritoneal dissemination involving small lesions. For late-phase peritoneal dissemination, a combination of ^64^Cu-ipRIT and PET-guided surgery with a single administration of ^64^Cu-labeled cetuximab was used by employing ^64^Cu-ipRIT to treat small lesions for down-staging and subsequent PET-guided surgery for resecting large tumor masses. In this study, we examined the effectiveness of integrated ^64^Cu therapy with early- and late-phase peritoneal-dissemination mouse models, which were generated by ip injection of tumor cells at 1 and 3–4 weeks before treatment, respectively. We found that the integrated ^64^Cu therapy with cetuximab effectively inhibited tumor growth and prolonged survival when treating early- and late-phase peritoneal dissemination in the mouse models. These findings suggested that integrated ^64^Cu therapy can serve as a novel treatment strategy against peritoneal dissemination.

## RESULTS

### Accumulation of the ip-injected ^64^Cu-labeled anti-EGFR antibody cetuximab in peritoneal dissemination

To verify the effectiveness of integrated ^64^Cu therapy, we generated early- and late-phase peritoneal-dissemination mouse models with human colon cancer HCT116 cells stably expressing red fluorescent protein (HCT116-RFP) by injecting the cells ip at 1 and 4 weeks before treatment, respectively. ^64^Cu was produced using a cyclotron, and the anti-EGFR antibody cetuximab was ^64^Cu-labeled using the chelator 3,6,9,15-tetraazabicyclo[9.3.1]pentadeca-1(15),11,13-triene-3,6,9-triacetic acid (PCTA). The resultant ^64^Cu-PCTA-cetuximab showed specific binding with high affinity to HCT116-RFP cells expressing EGFR (Supplementary Figures 1, 2). We compared the time-dependent accumulation of ^64^Cu-PCTA-cetuximab after ip or iv injection of small tumors in the early-phase peritoneal-dissemination mouse model (Figure 1B, 1C). ^64^Cu-PCTA-cetuximab rapidly accumulated at higher levels in small, intraperitoneal tumors after ip injection versus iv injection (Figure 1C). Analysis of the areas under the time–activity curves revealed that tumors accumulated 2.5-fold more ^64^Cu-PCTA-cetuximab after ip injection than after iv injection (*P* < 0.05) (Figure 1C).

### Distribution and safety of ip-injected ^64^Cu-PCTA-cetuximab

A biodistribution study was performed with tumor-free mice to examine the distribution of ip- or iv-injected ^64^Cu-PCTA-cetuximab to normal organs (Supplementary Figure 3A, 3B). Following ip injection, the radioactivity in ascites fluid was high at early time points (up to 6 h), and rapid clearance from the peritoneal cavity was observed thereafter. The radioactivity in other organs was low after both ip and iv injection. We examined hematological and biochemical parameters in tumor-free mice that received therapeutic doses of ^64^Cu-PCTA-cetuximab via ip or iv injection. Supplementary Figure 3C, 3D show the numbers of blood cells that were counted, including white blood cells (WBCs), red blood cells (RBCs), and platelets (PLTs). Both ip and iv injection of 37 MBq ^64^Cu-PCTA-cetuximab showed significant reductions in the blood cell counts. The ip injection of 22.2 MBq ^64^Cu-PCTA-cetuximab did not significantly reduce any blood cell numbers, whereas iv injection of 22.2 MBq ^64^Cu-PCTA-cetuximab significantly reduced the number of WBCs. Based on this finding, we used 22.2 MBq of ^64^Cu-PCTA-cetuximab as a therapeutic dose for ip injection with mice in this study. No significant differences were observed in any biochemical parameters, including glutamate oxaloacetate transaminase, glutamate pyruvate transaminase, and alkaline phosphatase activities measured to study liver function; urea nitrogen and creatinine levels measured to assess kidney function; or amylase and lipase activities determined to assess pancreas function, when compared to control ip- and iv-treated mice (Supplementary Figures 4, 5). Dosimetry analysis was conducted based on the biodistribution data with OLINDA/EXM software, which can estimate organ-absorbed doses in humans after administration of radiopharmaceuticals with the energies of photons and particles emitted from radionuclides [31]. The estimated absorbed doses to the pancreas and large intestine were relatively high in ip-injected mice (0.0456 mSv/MBq and 0.0384–0.0377 mSv/MBq, respectively), compared to those levels in iv-injected mice (Supplementary Tables 1, 2). However, the human radiation doses in these organs, which were estimated using the administration dose based on body weight, were sufficiently low relative to the reported tolerance doses (Supplementary Table 3).

### Efficacy of ^64^Cu-ipRIT against early-phase peritoneal dissemination

**Figure 2:**
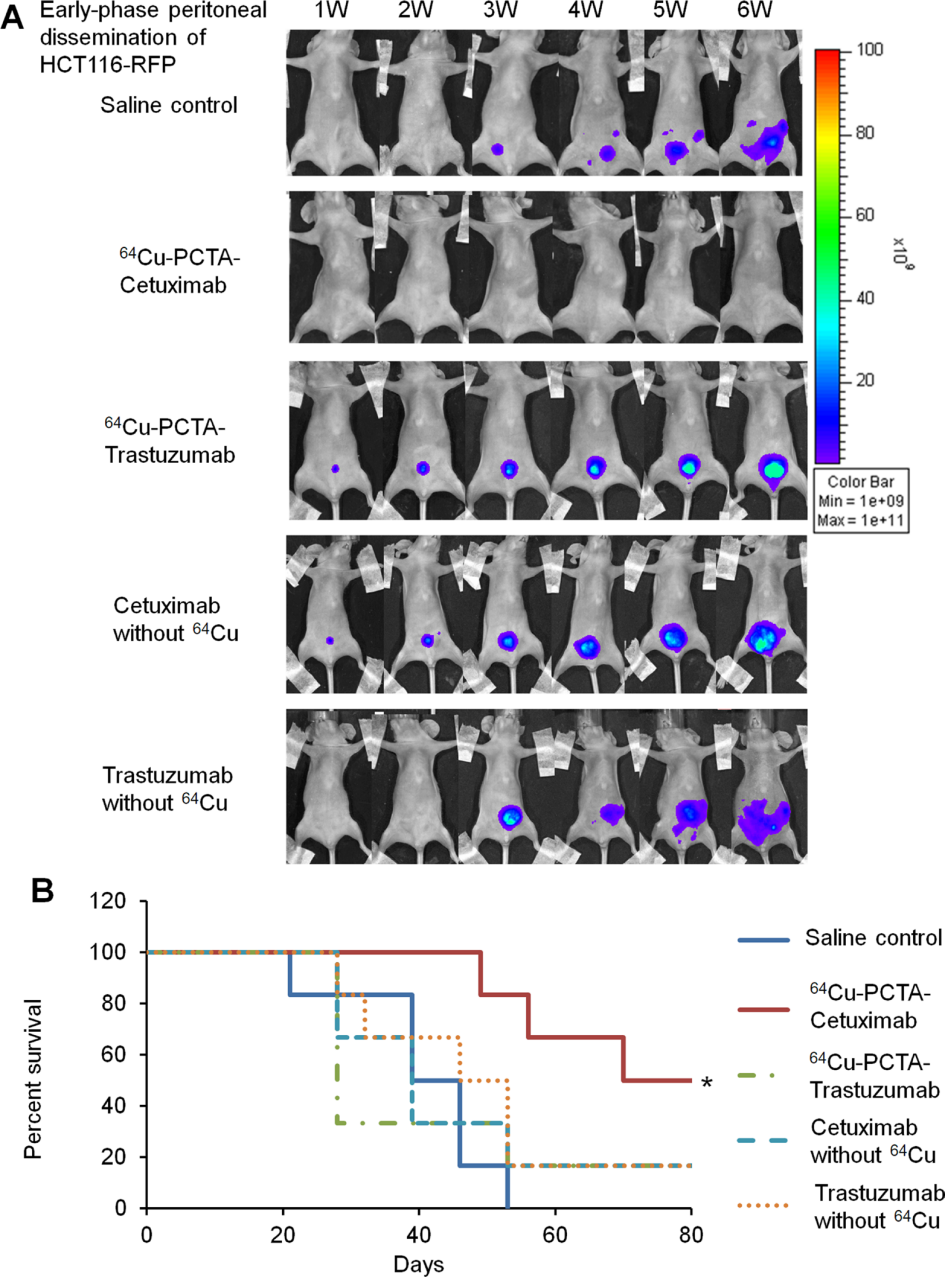
^64^Cu-ipRIT in an early-phase peritoneal dissemination model with HCT116-RFP cells (**A**) Representative images of mice treated with ^64^Cu-ipRIT against early-phase peritoneal dissemination of HCT116-RFP cells (saline control, ^64^Cu-PCTA-cetuximab, ^64^Cu-PCTA-trastuzumab, cetuximab without ^64^Cu, and trastuzumab without ^64^Cu groups). Tumor growth was observed by *in vivo* fluorescence imaging. (**B**) Survival curves (*n* = 6). ^*^P < 0.05 vs. saline control (log-rank test)

First, we investigated the efficacy of ^64^Cu-ipRIT with ^64^Cu-PCTA-cetuximab in treating early-phase peritoneal dissemination, using a mouse model generated with HCT116-RFP cells. ^64^Cu-PCTA-cetuximab (22.2 MBq) was injected ip into mice. For comparison purposes, we separately administered saline (control), ^64^Cu-PCTA-trastuzumab (22.2 MBq), and cetuximab and trastuzumab without ^64^Cu (5 mg/kg, twice a week for 80 days, for molecularly targeted antibody therapy). Trastuzumab was selected as a negative antibody because of its low binding affinity to HCT116-RFP cells (Supplementary Figure 6), which express low levels of HER2 (Supplementary Figure 2). ^64^Cu-PCTA-cetuximab inhibited tumor growth (Figure 2A) and extended survival significantly, compared with the saline control (*P* < 0.05) (Figure 2B). The other treatment groups (^64^Cu-PCTA-trastuzumab, cetuximab without ^64^Cu, and trastuzumab without ^64^Cu) showed little tumor growth inhibition (Figure 2A), and no significant difference in survival was observed versus the saline control (Figure 2B). Significant differences in survival were noted between the ^64^Cu-PCTA-cetuximab and other treatment groups (*P* < 0.05) (Figure 2B). Body weight loss >20% of the initial body weight was not observed in any treatment group up to the experimental endpoint. These results demonstrated that treatment with ^64^Cu-ipRIT with ^64^Cu-PCTA-cetuximab was effective in the early-phase peritoneal-dissemination model with HCT116-RFP tumor cells.

### Detection and resection of deeply located ip tumors using PET-guided surgery with ^64^Cu-PCTA-cetuximab

**Figure 3:**
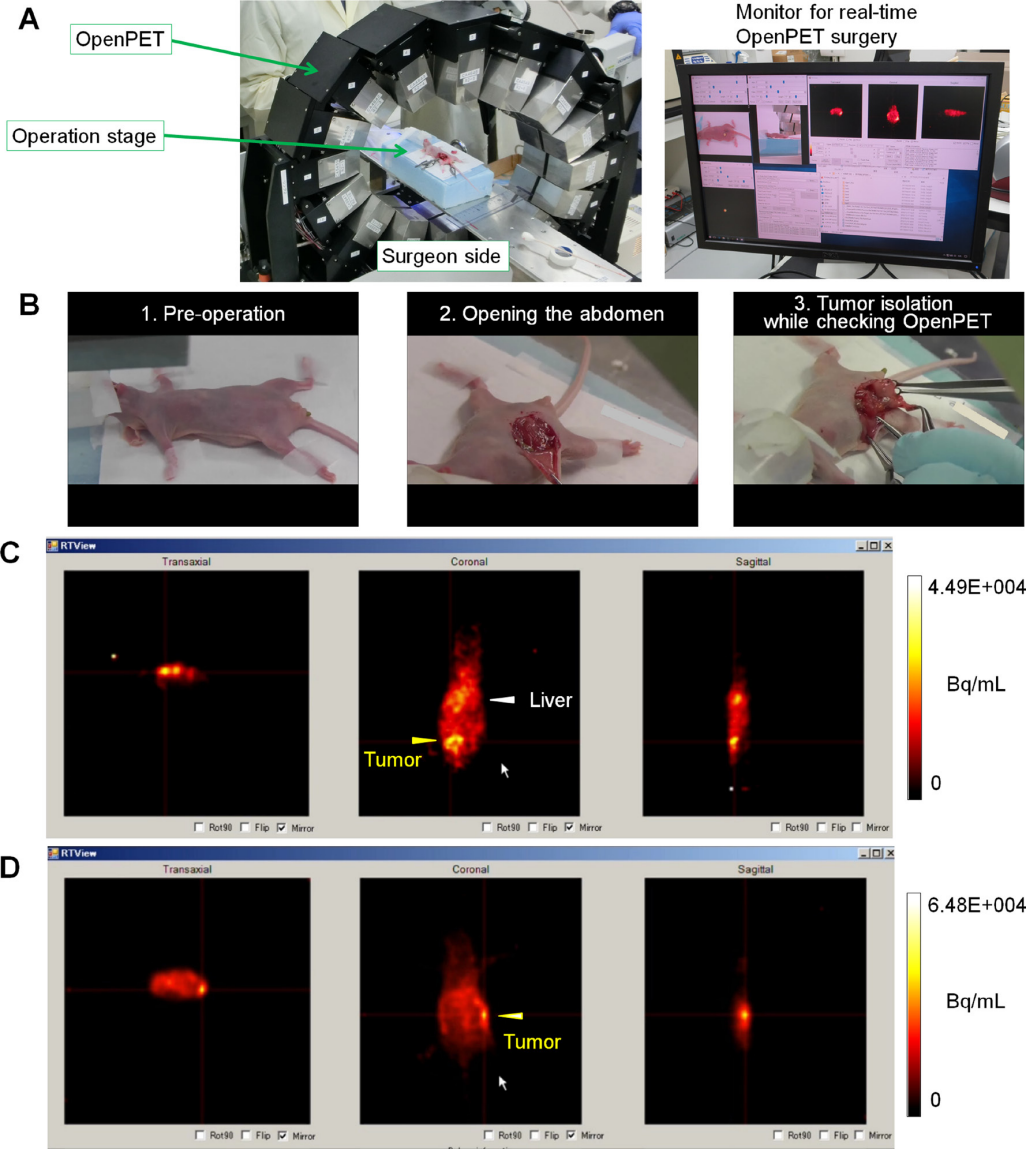
OpenPET-guided surgery (**A**) OpenPET system used for the surgical procedures. (**B**) Representative images of mice bearing transplanted HCT116-RFP tumors at deep sites during OpenPET-guided surgery. A series of images taken during OpenPET-guided surgery is shown, including pre-operation (left), opening the abdomen (middle), and tumor isolation while checking real-time OpenPET images (right) (see details in Supplementary Video 1). (**C**, **D**) OpenPET images. (C) A tumor (approximately 10 mm) located behind the intestine in the middle of peritoneal cavity is shown. (D) A small (3-mm) tumor located deeply in the peritoneal cavity is shown.

**Figure 4:**
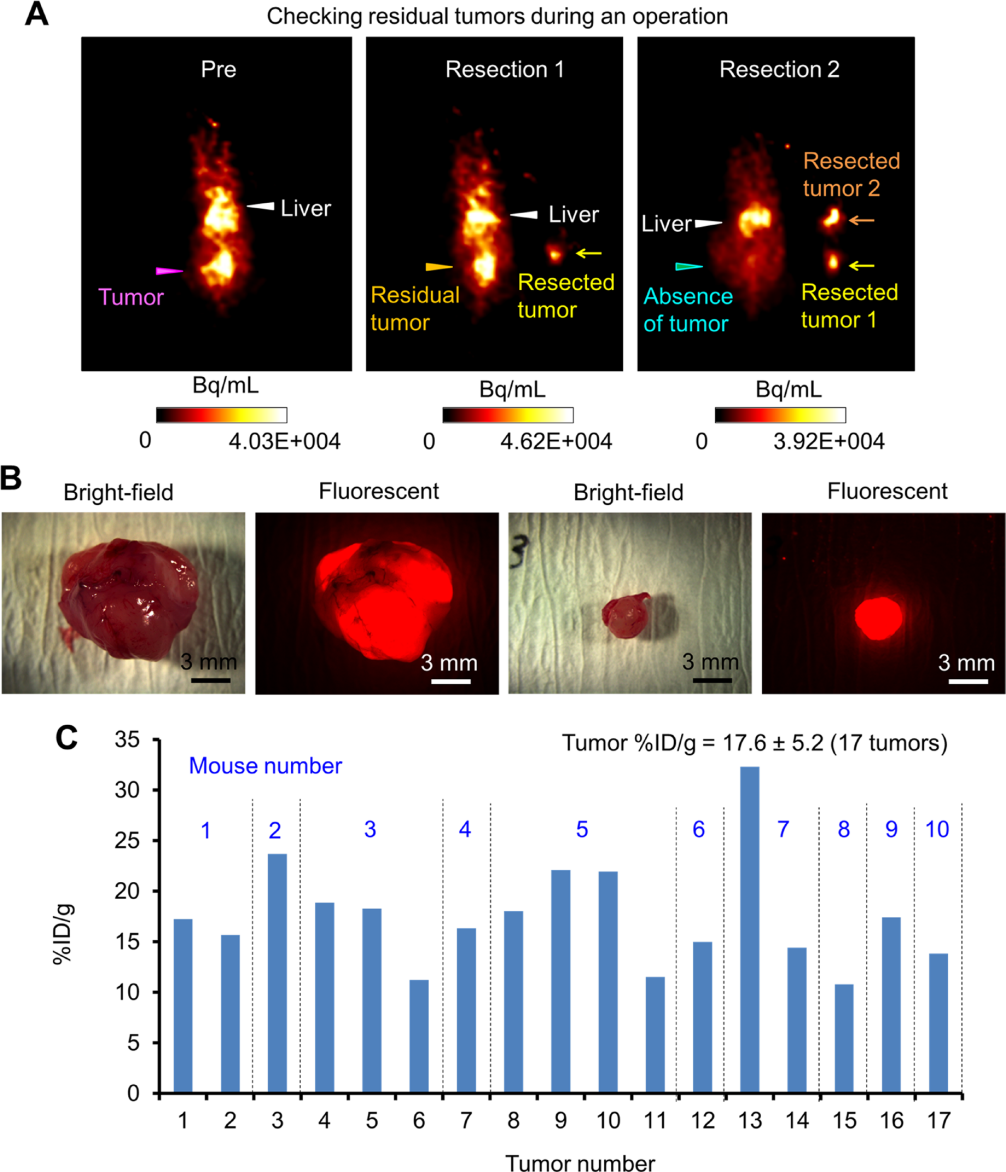
Tumor resection during OpenPET-guided surgery (**A**) A series of OpenPET images taken during tumor resection in OpenPET-guided surgery using mice bearing transplanted HCT116-RFP tumors. These images were obtained before the operation (left), after the first tumor resection (middle), and after the second tumor resection (right). After the first resection, a residual tumor was found by OpenPET. After the second tumor resection, no tumor signals were observed by OpenPET. (**B**) Bright-field and fluorescence images of resected tumors observed with a stereoscopic fluorescence microscope (10-mm and 3-mm tumors, left and right images). (**C**) The uptake of ^64^Cu-PCTA-cetuximab by 17 tumors resected from 10 different mice during OpenPET-guided surgery (%ID/g). The average %ID/g was 17.6 ± 5.2.

To assess the feasibility of using PET-guided surgery with ^64^Cu-PCTA-cetuximab to detect and remove deeply located ip tumors, OpenPET-guided surgery (Figure 3A) was performed with mice bearing HCT116-RFP tumors transplanted with Matrigel at deep sites in the peritoneal cavity. The mice were ip-administered ^64^Cu-PCTA-cetuximab (7.4 MBq/mouse, for imaging) 24 h before OpenPET-guided surgery. During OpenPET-guided surgery, tumors were clearly detected behind several organs in the peritoneal cavity (Figure 3B, 3C; Supplementary Video 1). The measurement time required to accumulate sufficient data to identify tumors was approximately 10–30 s. Tumors (approximately 10 mm in diameter) located deep inside the peritoneal cavity could be resected by monitoring during OpenPET surgery with real-time imaging (Figure 3B, 3C; Supplementary Video 1). These tumors could not be visually identified without OpenPET (Figure 3B). Moreover, the fluorescence of these tumors was not detected, since they were located behind the intestines (Supplementary Figure 7). It is worth noting that we successfully detected and resected deeply located, small (3-mm) tumors using this system (Figure 3D, Supplementary Video 2). OpenPET was useful in confirming the presence or absence of residual tumors during the operations (Supplementary Video 2) (Figure 4A). All resected tissues were confirmed to be tumors by detecting RFP signals after resection (3–12 mm) (Figure 4B). The uptake of ^64^Cu-PCTA-cetuximab in resected tumors was 17.6 ± 5.2 in terms of the percentage of injected dose per gram (%ID/g) (17 tumors from 10 mice) (Figure 4C). Using a tumor-free mouse, we confirmed that less background signal was present in the peritoneal cavity during OpenPET imaging after ip administration of ^64^Cu-PCTA-cetuximab (7.4 MBq/mouse, for imaging), although the liver showed physiological accumulation of ^64^Cu-PCTA-cetuximab (Supplementary Figure 8).

### Combination of ^64^Cu-ipRIT and PET-guided surgery for treating late-phase peritoneal dissemination

**Figure 5:**
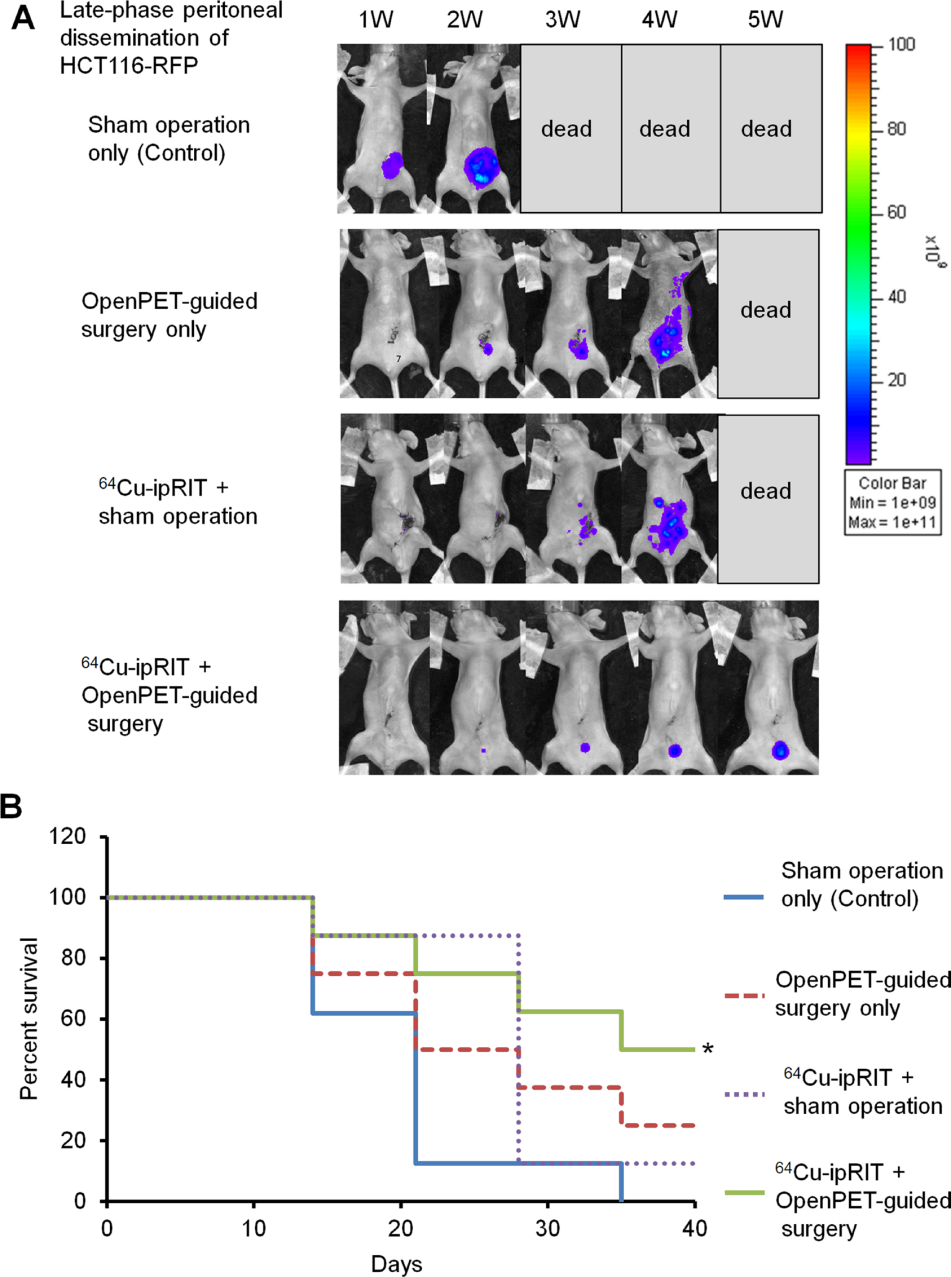
Combination of ^64^Cu-ipRIT and OpenPET-guided surgery in a late-phase peritoneal-dissemination model with HCT116-RFP cells (**A**) Representative images of mice in an *in vivo*-treatment study with a late-phase peritoneal-dissemination model generated with HCT116-RFP cells (sham operation-only control, OpenPET-guided surgery only, ^64^Cu-ipRIT + sham operation, or ^64^Cu-ipRIT + OpenPET-guided surgery). Tumor growth was observed by *in vivo* fluorescence imaging. (**B**) Survival curves (*n* = 8). ^*^P < 0.05 vs. sham operation-only control (log-rank test)

The usefulness of combining ^64^Cu-ipRIT and OpenPET-guided surgery was investigated with late-phase peritoneal dissemination in the mouse model with HCT116-RFP tumor cells. Employing ^64^Cu as a theranostic nuclide, both ^64^Cu-ipRIT and OpenPET-guided surgery can be performed with a single administration of ^64^Cu-PCTA-cetuximab. First, we injected a therapeutic dose of ^64^Cu-PCTA-cetuximab (22.2 MBq/mouse, therapeutic dose) ip into mice for down-staging with ^64^Cu-ipRIT, and then we conducted OpenPET-guided surgery at 48 h after the administration when the radioactivity had decayed to imaging levels. For comparison, we treated mice with ^64^Cu-ipRIT + a sham operation, OpenPET-guided surgery only, or a sham operation-only control. The combination of ^64^Cu-ipRIT and OpenPET-guided surgery inhibited tumor growth (Figure 5A) and extended survival significantly compared with the sham operation-only control (*P* < 0.05) (Figure 5B). OpenPET-guided surgery alone and ^64^Cu-ipRIT with a sham operation appeared to slightly extend survival, but the differences were not significant compared with the sham operation-only control (Figure 5B). No body weight loss >20% of the initial body weight was observed in any treatment group up to the experimental endpoint.

### Efficacy of integrated ^64^Cu therapy for treating early- and late-phase peritoneal-dissemination models of gastric cancer

**Figure 6:**
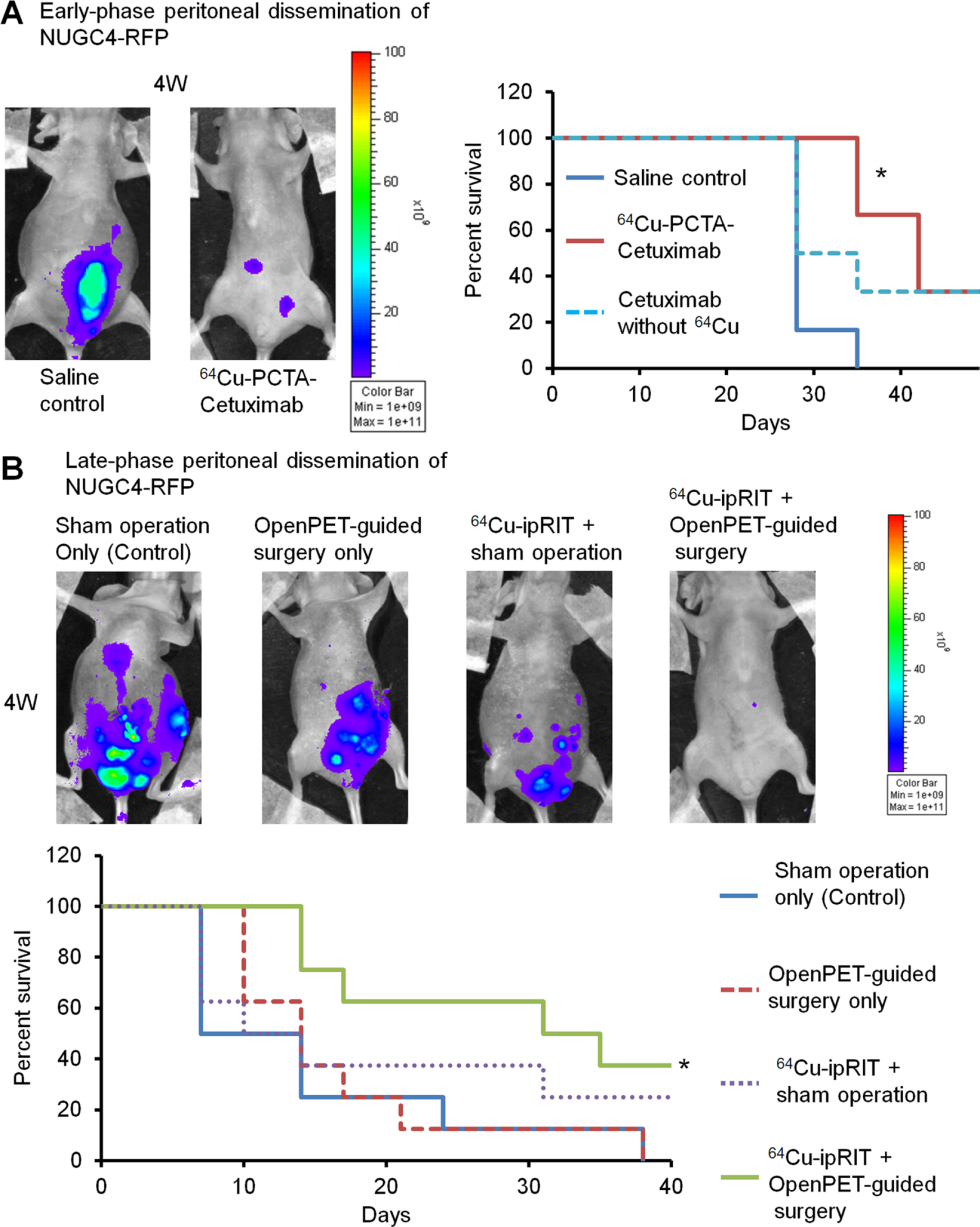
Integrated ^64^Cu therapy for peritoneal-dissemination models with NUGC4-RFP cells (**A**) The *in vivo* study of ^64^Cu-ipRIT with an early-phase peritoneal-dissemination model with NUGC4-RFP cells (saline control, ^64^Cu-PCTA-cetuximab, cetuximab without ^64^Cu). Representative images of mice observed by *in vivo* fluorescence imaging in the saline-control and ^64^Cu-PCTA-cetuximab groups at 4 weeks after treatment (left). Survival curves (*n* = 6) (right). ^*^P < 0.05 vs. saline control (log-rank test). (**B**) The *in vivo* study of combined treatment with ^64^Cu-ipRIT and OpenPET-guided surgery with a late-phase peritoneal-dissemination model using NUGC4-RFP cells (sham operation-only control, OpenPET-guided surgery only, ^64^Cu-ipRIT + sham operation, or ^64^Cu-ipRIT + OpenPET-guided surgery). Representative images of mice observed by *in vivo* fluorescence imaging in different groups (sham operation-only control, OpenPET-guided surgery only, ^64^Cu-ipRIT + sham operation, and ^64^Cu-ipRIT + OpenPET-guided surgery) at 4 weeks after treatment (upper). Survival curves (*n* = 8) (lower). ^*^P < 0.05 vs. sham operation-only control (log-rank test)

In addition to colorectal cancer cells, human gastric cancer NUGC4-RFP cells that overexpressed EGFR [32] were used to investigate the efficacy of integrated ^64^Cu therapy with ^64^Cu-PCTA-cetuximab. Early- and late-phase peritoneal-dissemination mouse models of NUGC4-RFP cells were generated by ip injection of cells at 1 and 3 weeks before treatment, respectively. For the early-phase peritoneal-dissemination model with NUGC4-RFP cells, ^64^Cu-ipRIT with ^64^Cu-PCTA-cetuximab inhibited tumor growth (Figure 6A, left panel) and extended survival significantly compared with the saline control (*P* < 0.05) (Figure 6A, right panel). No significant difference in survival was observed after cetuximab treatment without ^64^Cu compared with the saline control, although survival tended to be prolonged (Figure 6A, right panel). In the late-phase peritoneal-dissemination mouse model with NUGC4-RFP cells, combined ^64^Cu-ipRIT and OpenPET-guided surgery inhibited tumor growth (Figure 6B, upper panel) and prolonged survival significantly compared with the sham operation-only control (*P* < 0.05) (Figure 6B, lower panel). OpenPET-guided surgery alone and ^64^Cu-ipRIT + sham operation showed a tendency towards prolonged survival, but no significant differences in survival were found compared with the sham operation-only control (Figure 6B, lower panel). The level of ^64^Cu-PCTA-cetuximab uptake by the resected tumors was 14.1 ± 4.0 %ID/g (13 tumors from 8 mice). No body weight loss >20% of the initial body weight was observed in any treatment groups up to the experimental endpoint.

## DISCUSSION

In this study, we developed an integrated ^64^Cu therapy as a novel strategy to treat early- and late-phase peritoneal dissemination using ^64^Cu-ipRIT, or in combination with PET-guided surgery using the ^64^Cu-labeled anti-EGFR antibody cetuximab. It is difficult to treat peritoneal dissemination of tumor cells by conventional therapy, and the development of alternative treatment strategies to manage different phases of progression is needed [1]. We demonstrated that ^64^Cu-ipRIT inhibited tumor expansion and prolonged survival during early-phase peritoneal dissemination, which involves small tumors and tumor cells that likely spread in the peritoneal cavity. For late-phase peritoneal dissemination, which involves the coexistence of small and large tumor masses, the combination of ^64^Cu-ipRIT and OpenPET-guided surgery significantly inhibited tumor expansion and extended survival, whereas single treatment with either ^64^Cu-ipRIT or OpenPET-guided surgery was not significantly effective. These findings indicated that the integrated ^64^Cu therapy could be useful for treating various phases of peritoneal dissemination by selecting ^64^Cu-ipRIT or combination therapy with ^64^Cu-ipRIT and PET-guided surgery, depending on the extent of disease progression. Particularly, with late-phase peritoneal dissemination, the combination of ^64^Cu-ipRIT (for down-staging by treating small lesions) and PET-guided surgery (for resecting large tumor masses) would be beneficial for prolonging survival, and this method could serve as a novel treatment option to improve survival in late-phase peritoneal dissemination, which presently has no effective treatment.

ipRIT has been regarded as a potential option for treating small lesions in peritoneal dissemination. Tumor-specific binding antibodies labeled with the β^−^ emitters ^131^I (half-life = 8 days) and ^177^Lu (half-life = 6.7 days), or the alpha emitter ^225^Ac (half-life = 10 days) have been used to treat small lesions in early-phase peritoneal dissemination [4–6]. In this study, we demonstrated that ^64^Cu (half-life = 13 h), which emits β^−^ particles and Auger electrons, was also useful for ipRIT. We found that ip-injected ^64^Cu-PCTA-cetuximab showed high and rapid accumulation in small tumors in an early-phase peritoneal dissemination mouse model and that tumor accumulation of ^64^Cu-PCTA-cetuximab plateaued at 3 h after ip injection. These findings suggested that ^64^Cu can effectively target peritoneal dissemination by rapid accumulation after ip injection. Considering the relative ease of production and the availability of ^64^Cu for therapeutic usage [19, 22], ^64^Cu-ipRIT can potentially serve as an alternative, practical option to ipRIT for treating small metastases. Effective procedures for intraperitoneal administration of antibodies labeled with other radionuclides have been reported in clinical studies [33, 34], which suggests that ip administration of ^64^Cu-labeled antibody to humans is also feasible. Nonetheless, further preclinical and clinical studies to optimize an administration dose (as well as multiple dosages) for ^64^Cu-ipRIT should be performed to improve the effectiveness and outcome of this therapy.

In this study, ^64^Cu-ipRIT with ^64^Cu-PCTA-cetuximab targeting EGFR was effective in the early-phase peritoneal-dissemination model with HCT116-RFP and NUGC4-RFP cells. Overexpression of EGFR, sometimes accompanied by gene amplification, is observed in a wide variety of cancers including gastrointestinal cancers [26, 35–37]. Cetuximab has been reported to show high binding affinity for EGFR highly expressed in cancer cells, provided that EGFR has no ectodomain mutations preventing cetuximab binding, although EGFR ectodomain mutations are rarely observed in patients' gastrointestinal cancers [26, 36, 38]. Previous studies have reported that HCT116 and NUGC4 cells show high EGFR expression without ectodomain mutations [32, 39, 40], and it is therefore reasonable that ^64^Cu-ipRIT with ^64^Cu-PCTA-cetuximab was effective against the HCT116-RFP and NUGC4-RFP cells used in this study.

Using the early-phase peritoneal-dissemination model with HCT116-RFP cells, we demonstrated that ip injection of ^64^Cu-PCTA-cetuximab, showing high binding affinity to HCT116-RFP cells, was effective. In contrast, ip injection of ^64^Cu-PCTA-trastuzumab, showing low binding affinity to HCT116-RFP cells, was ineffective. These results suggested that using antibodies with high binding affinity to the target tumor cells is necessary in ^64^Cu-ipRIT and, therefore, that checking the antigen expression or binding affinity of the antibodies used before therapy would be clinically beneficial. In experiments with the early-phase peritoneal-dissemination model using HCT116-RFP cells, we demonstrated that ^64^Cu-ipRIT with ^64^Cu-PCTA-cetuximab was effective, while molecularly targeted therapy with unlabeled cetuximab was ineffective. In the early-phase peritoneal-dissemination model with NUGC4-RFP cells, ^64^Cu-ipRIT with ^64^Cu-PCTA-cetuximab was also effective, and molecularly targeted therapy with cetuximab alone was slightly effective. It has been reported that mutations in the KRAS gene represent one cause of the ineffectiveness of targeted therapy with cetuximab [41] and that HCT116 cells possess a KRAS mutation [42], but that NUGC4 cells do not [43]. It is therefore reasonable that cetuximab therapy without ^64^Cu was ineffective against HCT116-RFP cells and slightly effective against the NUGC4-RFP cells in this study. Our results also suggested that ^64^Cu-PCTA-cetuximab could be used to treat peritoneal dissemination of tumor cells expressing EGFR, with or without a KRAS mutation.

In this study, we focused on ^64^Cu because it can be used for simultaneous imaging and therapy. This feature of ^64^Cu facilitates PET-guided surgery with ^64^Cu-ipRIT after a single administration of the ^64^Cu-labeled antibody. We demonstrated that PET-guided surgery with ^64^Cu-PCTA-cetuximab using the OpenPET system can be used to detect and resect deeply located intraperitoneal tumors, which can be difficult to find with the naked eye or with fluorescent imaging. In addition, PET-guided surgery with ^64^Cu-PCTA-cetuximab could identify residual tumors during operation. These results provided a rationale for using PET-guided surgery with ^64^Cu-PCTA-cetuximab as a helpful tool for surgical operations against tumors in the peritoneal cavity. We also observed that the combination of ^64^Cu-ipRIT and PET-guided surgery effectively inhibited tumor growth and prolonged survival in the late-phase peritoneal-dissemination model, whereas a single treatment with OpenPET-guided surgery or ^64^Cu-ipRIT was less effective than combination therapy. The radiation range of ^64^Cu in tissue, which is reported to be less than 1.4 mm [44], could explain the limitation of ^64^Cu-ipRIT for the larger tumor masses. These findings suggest that PET-guided surgery, in combination with ^64^Cu-ipRIT, would be beneficial for improving survival in late-phase peritoneal dissemination by removing large tumor masses that are too large to be treated by ^64^Cu-ipRIT.

The concept of real-time, image-guided surgery has been considered promising for improving the results of surgical operations for peritoneal dissemination. Therefore, real-time, fluorescence image-guided surgery has been well studied and shown to be effective for detecting surface small tumors that are invisible to the naked eye [45]. However, detecting tumors located deeply behind other organs is difficult due to the short range of fluorescent signals [45]. In this study, we showed that PET-guided surgery with the OpenPET-guided system was useful for detecting and resecting tumors deeply located in the peritoneal cavity. In the future, multimodal real-time surgery based on PET and fluorescence imaging (using the advantages of both approaches) might be promising for surgical treatment of peritoneal dissemination. Radiation exposure to surgeons during PET-guided surgery may be raised as a controversial issue. To address this problem, a combination of robotic surgery with real-time PET guidance should be considered in the future. In this study, we used a small-sized OpenPET system to verify the feasibility of PET-guided surgery in mice. Recently, a large-sized OpenPET system for human use was developed in our institute [46], and the feasibility of OpenPET-guided surgery in clinical settings will be investigated in the near future.

This study has several limitations. We designed this animal study to evaluate the efficacy and toxicity of integrated ^64^Cu therapy with ^64^Cu-PCTA-cetuximab. However, the mouse models of peritoneal dissemination implemented in this study might be too simple to represent the human disease in terms of the size and distribution of tumors. In addition, discrepancies in immunoreactivity of ^64^Cu-PCTA-cetuximab might be observed between mice and humans. Therefore, the efficacy, biodistribution, and toxicity of ip-administered ^64^Cu-PCTA-cetuximab should be carefully evaluated in future clinical studies in humans.

In this study, we developed an integrated ^64^Cu therapy to treat early- and late-phase peritoneal dissemination using ^64^Cu-ipRIT or combining it with PET-guided surgery. Considering the future clinical development of this approach, monitoring the disease status to select ^64^Cu-ipRIT or combination therapy with ^64^Cu-ipRIT and PET-guided surgery is important; methods to detect early-phase peritoneal dissemination (which is difficult with current technologies) are particularly needed. Recently, liquid biopsy methods have been developed to detect early-phase peritoneal dissemination [47] and these methods may contribute to overcoming this problem. If liquid biopsies could indicate the presence of peritoneal dissemination, diagnosis with OpenPET would be then necessary to determine the additional use of PET-guided surgery with ^64^Cu-ipRIT.

In conclusion, we developed a novel therapeutic strategy, integrated ^64^Cu therapy, to treat early- and late-phase peritoneal dissemination—either by ^64^Cu-ipRIT or combining it with PET-guided surgery—using the theranostic agent ^64^Cu-PCTA-cetuximab. ^64^Cu-ipRIT was effective for treating early-phase peritoneal dissemination, and the combination of ^64^Cu-ipRIT and PET-guided surgery was beneficial for late-phase peritoneal dissemination in mice. Because the production of ^64^Cu is feasible, integrated ^64^Cu therapy can potentially serve as a practical tool for treating peritoneal dissemination.

## MATERIALS AND METHODS

### Cell culture and mice

Human colon cancer HCT116 cells (CCL-247; American Type Cell Collection) stably expressing RFP (HCT116-RFP), established in our previous study [48], were used. Human gastric cancer NUGC4-RFP cells (Anticancer) were also used in this study. Cells were cultured in a humidified atmosphere of 5% CO_2_ at 37°C. DMEM and RPMI 1640 media (Wako) supplemented with 10% fetal bovine serum were used as growth medium for HCT116-RFP and NUGC4-RFP cells, respectively. Exponentially growing cells were detached from the culture plates with trypsin and used in this study. The number of viable cells was determined using the Trypan blue dye-exclusion method. All cell lines were mycoplasma-free.

Six-week-old female BALB/c nude mice (15–20 g body weight) were obtained from Japan SLC and used in this study. Before the experiments, the mice were acclimated for at least 1 week. All animal experimental procedures were approved by the Animal Ethics Committee of the National Institutes for Quantum and Radiological Science and Technology (QST, Chiba, Japan) and conducted in accordance with the institutional guidelines. To generate early- and late-phase peritoneal-dissemination mouse models of HCT116-RFP, 5 × 10^5^ cells suspended in 500 μL phosphate-buffered saline (PBS) were injected ip at 1 and 4 weeks before treatment, respectively. NUGC4-RFP cells (5 × 10^6^) were injected in the same manner at 1 and 3 weeks before treatment for the early- and late-phase models, respectively.

### Antibody labeling with ^64^Cu

The anti-human EGFR monoclonal antibody cetuximab (Merck Serono) was used in this study. ^64^Cu was produced using a cyclotron and purified according to previously reported procedures [22]. For antibody conjugation, the bifunctional chelator p-SCN-Bn-PCTA (Macrocyclics) was used. Antibody conjugation and ^64^Cu labeling were accomplished using previously described methods, with slight modifications [27, 49, 50], with slight modifications. Briefly, for antibody conjugation, a cetuximab solution (2 mg/ml in 50 mM borate buffer, pH 8.5) was prepared by buffer exchange with a Vivaspin ultrafiltration device (Sartorius). PCTA was dissolved in DMSO and added to the cetuximab solution (2 mg/ml) at chelate to antibody molar ratio of 5:1. To obtain PCTA-conjugated cetuximab, the mixtures were incubated overnight at 37°C. We determined that 3 PCTA molecules were conjugated to the antibody, using a reported method [49]. For ^64^Cu labeling, a solution of PCTA-conjugated cetuximab was prepared at 2 mg/ml in 0.1 M ammonium citrate buffer (pH 5.5). ^64^CuCl_2_ was produced and purified according to previously reported procedures [17, 22], and a ^64^CuCl_2_ solution (370 MBq in 300 μL 0.1 M ammonium citrate buffer, pH 5.5) was prepared. The resultant ^64^CuCl_2_ solution was added to the PCTA-conjugated cetuximab solution (2 mg/ml) at a 3:1 ratio (vol:vol) and incubated for 1 h at 40°C. The radiochemical purity of ^64^Cu-PCTA-cetuximab was determined by radio-thin-layer chromatography. The ^64^Cu-PCTA-cetuximab was obtained at high radiolabeling yield and radiochemical purity (>95%), and the specific activity was 1.1 to 1.7 GBq/mg, which was determined using previously described methods [49]. Cell-binding and competitive-inhibition assays were performed with HCT116-RFP cells, as previously described [49] (see Supplementary Methods for details). An anti-human HER2 monoclonal antibody trastuzumab was obtained from Chugai Pharmaceutical and used in this study for comparison purposes. Trastuzumab was labeled with ^64^Cu in a similar manner to cetuximab, and the radiochemical purity and specific activity of the resultant ^64^Cu-PCTA-trastuzumab were similar to the values for ^64^Cu- PCTA-cetuximab. In the animal experiments using ^64^Cu-labeled antibody, the injected protein dose was adjusted to 20 μg per mouse by adding an unlabeled antibody.

### Distribution and toxicity of ^64^Cu-PCTA-cetuximab after ip or iv injection

The distribution of ^64^Cu-PCTA-cetuximab after ip or iv-injection was examined in small tumors (approximately 2-mm diameter) in the early-phase peritoneal-dissemination mouse model with HCT116-RFP cells and in normal organs using non-tumor bearing mice, respectively. Mice were injected ip or iv with a low dose of ^64^Cu-PCTA-cetuximab (3.7 MBq/mouse; *n* = 4/group) and sacrificed at 3, 6, 18, 24, or 48 h post-injection. For tumor distribution, tumors in the peritoneum were isolated under a fluorescent stereoscopic microscope (MZ16F, Leica), and densitometric analysis with autoradiography was performed to measure radioactivity in the small tumors using a previously reported method [17]. Briefly, the excised tumors were embedded in optimal cutting temperature compound (Tissue-Tek) and frozen on crushed dry ice. The tumors were sliced into 8-mm-thick sections with a cryostat (Leica). The frozen sections were subjected to autoradiography. Autoradiography images were obtained with the frozen sections by exposure to an imaging plate (BAS-MS 2040; Fuji Photo Films). The imaging plate was analyzed using a bioimaging analyzer (FLA-7000; Fuji Photo Films). A calibration curve correlating the photostimulated luminescence intensity on the imaging plate to known ^64^Cu concentrations was also obtained in a similar manner and used to determine %ID/g uptake of tumors. We confirmed that the %ID/g values obtained by densitometric analysis were similar to those obtained for normal organs, based on the radioactivity per unit of organ weight, as described below.

For organ distribution, the organs of interest (liver, kidney, small intestine, large intestine, muscle, stomach, spleen, pancreas, heart, lung, bone, and the remainder of the body) were isolated, and blood, urine, and feces were also collected. The organ and blood samples were weighed. Radioactivity levels were measured with a γ-counter (1480 Automatic gamma counter Wizard 3; PerkinElmer). The biodistribution data were calculated as the %ID/g for the organs and blood and the %ID for the urine and feces, and dosimetry analysis was performed based on these data to estimate mean absorbed doses of ^64^Cu-PCTA-cetuximab (mSv/MBq) in humans. The mean %ID/g values were converted into corresponding human values, based on the standard body weights for mice (20 g) and humans (73.7 kg) [51]. These values were processed with OLINDA/EXM software [31], which used a dynamic bladder model with a voiding interval of 4.8 h and a peritoneal cavity model.

To evaluate side effects, hematological and biochemical parameters were measured in tumor-free mice that received 0, 22.2, or 37 MBq of ^64^Cu-PCTA-cetuximab via ip or iv injection (*n* = 4/group). Measurements of hematological parameters were performed on day 0 (just before ^64^Cu-PCTA-cetuximab injection) and on days 7, 14, 21, 28, and 35, using blood collected from the tail vein. The densities of WBCs, RBCs, and PLTs were determined using a hematological analyzer (Celltac MEK-6458, Nihon Kohden). Biochemical parameters were measured as described in the Supplementary Methods section.

### *In vivo*-treatment study of ^64^Cu-ipRIT using the early-phase peritoneal-dissemination model

The efficacy of ^64^Cu-ipRIT with ^64^Cu-PCTA-cetuximab for early-phase peritoneal dissemination was investigated. Using HCT116-RFP cells, mice with early-phase peritoneal dissemination were randomized into 5 groups (*n* = 6/group). Mice in the ^64^Cu-PCTA-cetuximab group were injected ip with ^64^Cu-PCTA-cetuximab (22.2 MBq, day 0). For comparison purposes, mice were examined after administration with saline (saline control group, day 0), ^64^Cu-PCTA-trastuzumab (22.2 MBq, day 0; ^64^Cu-PCTA-trastuzumab group), or cetuximab and trastuzumab without ^64^Cu (5 mg/kg, twice a week for 80 days; cetuximab and trastuzumab without ^64^Cu groups, respectively). Trastuzumab was selected as a negative-control antibody because of its low binding affinity to HCT116 cells. The doses of cetuximab and trastuzumab administered were selected based on previous reports [52, 53]. Mice were weighed and observed for 80 days, and tumor growth was monitored using *in vivo* fluorescent imaging with an IVIS Lumina imaging system (Caliper). Mice were sacrificed at a humane endpoint, which was defined as noticeable extension of the abdomen, development of ascites, or body weight loss (>20%). Treatment with NUGC4-RFP cells was performed similarly using mice administered ^64^Cu-PCTA-cetuximab (22.2 MBq), cetuximab without ^64^Cu (5 mg/kg, twice a week), or saline (saline control) (*n* = 6/group), with an observation period of 50 days.

### OpenPET-guided surgery

Mice bearing HCT116-RFP tumors at deep sites in the peritoneal cavity were used to examine the feasibility of OpenPET-guided surgery. For this experiment, HCT116-RFP cells (1 × 10^7^ cells) in 50 μl PBS were diluted in 50 μl Matrigel (BD Biosciences) and seeded into deep sites in the peritoneal cavity of each mouse. One week later, ^64^Cu-PCTA-cetuximab (7.4 MBq/mouse, for imaging) was administered ip into the mice. Twenty-four hours later, OpenPET-guided surgery was performed with a prototype of the OpenPET system, which was developed previously for use in small animal experiments [24, 25]. This system contains 32 detector blocks (4-layer depth-of-interaction detector having 16 × 16 × 4 crystals) in a cylinder with the diameter of 25 cm, and the detectors are axially shifted incrementally to form an accessible open space (14-cm wide) for surgical procedures with mice. The field of view (having a cylindrical shape cut by 2 parallel planes slanted at 45°relative to the axial direction) was 11.4 cm in diameter with a 10.2-cm axial length. The prototype had a spatial resolution of approximately 2 mm.

To accomplish real-time imaging during OpenPET-guided surgery, we used a 1-pass list-mode dynamic row-action maximum-likelihood algorithm with a graphics-processing unit for high-speed reconstruction, which enabled image updating in cycles of <1 s while accumulating list-mode data. The parameters used for OpenPET imaging were as follows: the voxel size was 1.5 mm; sensitivity and random corrections were applied; and absorption and scatter corrections were not applied. Reconstructed OpenPET images were represented as sliced images of the transaxial, coronal, and sagittal planes on the screen in front of the surgeon. OpenPET images were displayed as radioactivity-density values (kBq/mL) determined based on calibration with standards having known radioactivities. During surgery, the mice remained under 2% isoflurane anesthesia, and their body temperatures were maintained with a heater. Before beginning OpenPET-guided surgery, a surgeon checked the real-time OpenPET images to identify the locations of tumors in recipient mice, and then the abdominal wall and skin were cut to open the peritoneal cavity. Subsequently, a surgeon gently isolated tumors located in the peritoneal cavity, while checking the real-time OpenPET images. After resection, the signals of isolated tumors and the presence or absence of residual tumors were also examined with the OpenPET system. The fluorescent signals of the isolated tumors were also confirmed with a stereoscopic fluorescence microscope (MZ16F, Leica). The abdominal wall and skin were closed in 2 layers with surgical sutures. For some of the recipient mice, observation with a stereoscopic fluorescence microscope connected to the OpenPET system was performed to examine whether signals from tumors were detectable, as previously described [54, 55].

### ^64^Cu-ipRIT and OpenPET-guided surgery for the late-phase peritoneal-dissemination models

The efficacy of combining ^64^Cu-ipRIT and OpenPET-guided surgery was examined with mouse models of late-phase peritoneal dissemination, using HCT116-RFP and NUGC4-RFP cells. Mice with late-phase peritoneal dissemination were randomized into 4 groups for each cell line (*n* = 8/group). For combination treatment with ^64^Cu-ipRIT and OpenPET-guided surgery, a therapeutic dose of ^64^Cu-PCTA-cetuximab (22.2 MBq) was injected ip into mice for ^64^Cu-ipRIT, and OpenPET-guided surgery was performed at 48 h after administration, as described above (^64^Cu-ipRIT + OpenPET-guided surgery group). For comparison purposes, the ^64^Cu-ipRIT + sham operation, OpenPET-guided surgery-only, or sham operation-only (control) groups were also prepared. For the ^64^Cu-ipRIT + sham operation group, ^64^Cu-PCTA-cetuximab (22.2 MBq) was injected in a similar manner, and the sham operation (consisting of resecting only the tumors observed with the naked eye) was conducted without OpenPET observation. For the OpenPET-guided surgery-only group, mice were administered ip with an imaging dose of ^64^Cu-PCTA-cetuximab (7.4 MBq), and OpenPET-guided surgery was performed at 24 h after administration. For the sham operation-only control group, saline was administered instead of ^64^Cu-PCTA-cetuximab, and a sham operation was conducted as described above. Mice were weighed and observed for 40 days while monitoring tumor growth with a IVIS Lumina imaging system. Mice were sacrificed when reaching a humane endpoint, which was defined as a noticeable extension of the abdomen, development of ascites, or body weight loss (>20%).

### Statistical analysis

Data are expressed as the mean and standard deviation. *P* values were calculated using a 2-tailed *t*-test for comparisons between 2 groups or 1-way analysis of variance (ANOVA) for comparisons among multiple groups. Time–activity curves of tumor accumulation of ^64^Cu-PCTA-cetuximab were analyzed by 2-way ANOVA. Differences in survival were evaluated by the log-rank test. *P* values < 0.05 were considered statistically significant.

## SUPPLEMENTARY MATERIALS TABLES AND FIGURES






