# Supplementary material for: Integrated treatment using intraperitoneal radioimmunotherapy and positron emission tomography-guided surgery with 64Cu-labeled cetuximab to treat early- and late-phase peritoneal dissemination in human gastrointestinal cancer xenografts
Source: Oncotarget. 2018 Jun 22;9(48):28935–50. doi: 10.18632/oncotarget.25649 (PMC6034757; doi:10.18632/oncotarget.25649)
Supplement: Supplementary file 1 [file oncotarget-09-28935-s001.pdf]

Integrated treatment using intraperitoneal radioimmunotherapy and positron emission tomography-guided surgery with ^{64}Cu-labeled cetuximab to treat early- and late-phase peritoneal dissemination in human gastrointestinal cancer xenografts

SUPPLEMENTARY MATERIALS

Immunohistochemistry for EGFR and HER2 expression

Immunohistochemical analysis was performed with paraffin-embedded sections of HCT116-RFP spheroids cultured in 96-well nano-culture plates (SCIVAX Life Sciences), as previously described [1]. Briefly, the collected spheroids were embedded in iPGell (Genostaff, Tokyo, Japan), fixed with 10% buffered formalin (Genostaff) for 2 d at room temperature, processed for paraffin embedding, and sectioned according to standard histological procedures to obtain 6-µM sections. After deparaffinization and dehydration, the sections were treated by microwaving for antigen retrieval and 0.3% hydrogen peroxide in methanol for 30 min to quench endogenous peroxidases. To block nonspecific binding, a protein-blocking agent (G-Block, Genostaff) was applied for 10 min at room temperature. Endogenous avidin and biotin activities were blocked using the Avidin/Biotin Blocking Kit (Vector). The sections were incubated overnight at 4° C with a primary antibody against EGFR (1 µg/mL; CST) and HER2 (1 µg/mL; CST). After washing the sections with Tris-buffered saline (TBS), a biotinylated secondary antibody (E0432, Dako) was applied for 30 min at room temperature. After another TBS wash, the sections were incubated with streptavidin peroxidase reagent (426062, Nichirei) for 5 min and washed with TBS. Colour development was performed using 3,3′-diaminobenzidine tetrahydrochloride solution with H_2O_2, and sections were counterstained with hematoxylin.

Cell-binding and competitive-inhibition assays with ^{64}Cu-PCTA-cetuximab

Cell-binding and competitive-inhibition assays with ^{64}Cu-PCTA-cetuximab (synthesized in this study) were performed as previously described [2]. Briefly, for cell-binding assays, HCT116-RFP cells were serially diluted in PBS with 1% bovine serum albumin (BSA) (Sigma) and incubated with ^{64}Cu-PCTA-cetuximab on ice for 1 h. After washing, the radioactivity bound to the cells was measure using a γ-counter (1480 Automatic gamma counter Wizard 3; PerkinElmer). The immunoreactivity of ^{64}Cu-PCTA-cetuximab was determined by the Lindmo method [3]. For competitive-inhibition assays, HCT116-RFP cells were incubated with ^{64}Cu-PCTA-cetuximab and various concentrations of unlabelled cetuximab on ice for 1 h. After washing, the radioactivity bound to the cells was measure as described above. The dissociation constant was determined based on a competitive-inhibition assay. To compare the cell-binding affinities of ^{64}Cu-PCTA-cetuximab and ^{64}Cu-PCTA-trastuzumab, 1×10^6 cells diluted in PBS with 1% BSA were incubated with each ^{64}Cu-labeled antibody and the radioactivity was measured in a manner similar to that described above.

Measurement of biochemical parameters

Biochemical parameters were measured at day 35, using mouse plasma prepared with blood collected from the heart. Levels of glutamate oxaloacetate transaminase (GOT), glutamate pyruvate transaminase (GPT), and alkaline phosphatase (ALP) were measured to assess liver function; urea nitrogen (BUN) and creatinine (CRE) were measured for kidney function; and amylase (AMYL) and lipase (LIP) were measured for pancreas function, using a blood biochemical analyser (Dri-Chem 7000VZ, Fuji Film).

REFERENCES

1. Yoshii Y, Furukawa T, Waki A, Okuyama H, Inoue M, Itoh M, Zhang MR, Wakizaka H, Sogawa C, Kiyono Y, Yoshii H, Fujibayashi Y, Saga T. High-throughput screening with nanoimprinting 3D culture for efficient drug development by mimicking the tumor environment. Biomaterials. 2015; 51:278–289.
2. Sugyo A, Tsuji AB, Sudo H, Nagatsu K, Koizumi M, Ukai Y, Kurosawa G, Zhang MR, Kurosawa Y, Saga T. Evaluation of Zr-89-labeled human anti-CD147 monoclonal antibody as a positron emission tomography probe in a mouse model of pancreatic cancer. PLoS One. 2013; 8:e61230.
3. Lindmo T, Boven E, Cuttitta F, Fedorko J, Bunn PA. Determination of the immunoreactive fraction of radiolabeled monoclonal-antibodies by linear extrapolation to binding at infinite antigen excess. J Immunol Methods. 1984; 72:77–89.
4. ICRP, 2007. The 2007 Recommendations of the International Commission on Radiological Protection. ICRP Publication 103. Ann. ICRP 37. 2007; 2–4.
5. A Fauci EB, K Isselbacher, J Wilson, J Martin, D Kasper, S Hauser, D Longo. Harrison’s Principles of Internal Medicine 14th Edition. 1998:525–526.

Supplementary Video 1: OpenPET-guided surgery with ^{64}Cu-PCTA-cetuximab in the case of tumours located deeply in the peritoneal cavity. Video shows OpenPET-guided surgery detected tumours located deeply in the peritoneal cavity (approximately 10 mm) and the surgeon resected the tumours while monitoring it with the OpenPET real-time imaging. The tumours could not be visually identified by eye. See Supplementary_Video_1

Supplementary Video 2: OpenPET-guided surgery with ^{64}Cu-PCTA-cetuximab in the case of relatively small tumours located deeply in the peritoneal cavity. Video shows OpenPET-guided surgery detected and resected 3-mm size small tumours located deeply in the peritoneal cavity using OpenPET system. See Supplementary_Video_2

Supplementary Table 1: Mean estimated human absorbed doses of ^{64}Cu-PCTA-cetuximab following ip administration, extrapolated from mouse biodistribution data

Target organ	Total estimated absorbed dose (mSv/MBq)
Adrenals	0.0138
Brain	0.0113
Breasts	0.0106
Gallbladder wall	0.0148
Lower large intestinal wall	0.0384
Small intestine	0.0290
Stomach wall	0.0198
Upper large intestinal wall	0.0377
Heart wall	0.0348
Kidneys	0.0225
Liver	0.0321
Lungs	0.0119
Muscle	0.0076
Ovaries	0.0143
Pancreas	0.0456
Red marrow	0.0106
Osteogenic cells	0.0226
Skin	0.0096
Spleen	0.0351
Testes	0.0109
Thymus	0.0119
Thyroid	0.0112
Urinary bladder wall	0.0178
Uterus	0.0151
Total body	0.0137

Supplementary Table 2: Mean estimated human absorbed doses of ^{64}Cu-PCTA-cetuximab following iv administration, extrapolated from mouse biodistribution data

Target organ	Total estimated absorbed dose (mSv/MBq)
Adrenals	0.0171
Brain	0.0141
Breasts	0.0134
Gallbladder wall	0.0190
Lower large intestinal wall	0.0300
Small intestine	0.0300
Stomach wall	0.0222
Upper large intestinal wall	0.0299
Heart wall	0.0554
Kidneys	0.0328
Liver	0.0533
Lungs	0.0151
Muscle	0.0091
Ovaries	0.0164
Pancreas	0.0145
Red marrow	0.0129
Osteogenic cells	0.0282
Skin	0.0120
Spleen	0.0393
Testes	0.0135
Thymus	0.0153
Thyroid	0.0140
Urinary bladder wall	0.0188
Uterus	0.0165
Total body	0.0171

Supplementary Table 3: Comparison between the estimated and tolerance doses in critical organs during ^{64}Cu-PCTA-cetuximab therapy with ip or iv administration

	Organ*	Estimated dose in human (Sv/male)**	Tolerance dose of radiation (Sv)***
ip	Lower large intestinal wall	3.14	35–50
	Small intestine	2.37	40
	Upper large intestinal wall	3.08	35–50
	Kidneys	1.84	23
	Liver	2.62	30
	Pancreas	3.73	30
	Red marrow	0.86	2.5
iv	Lower large intestinal wall	2.46	35–50
	Small intestine	2.45	40
	Upper large intestinal wall	2.45	35–50
	Kidneys	2.69	23
	Liver	4.36	30
	Pancreas	1.19	30
	Red marrow	1.06	2.5

*Organs normally considered as those of concern in ^{64}Cu-PCTA-cetuximab therapy are listed. **Estimated doses with 81.8 GBq/adult man (73.7 kg), which were calculated from 22.2 MBq/mouse based on body weight, during ^{64}Cu-PCTA-cetuximab therapy. ***Reported tolerated doses of radiation in organs [4, 5]. The values were compared to the estimated doses in human organs in the text.

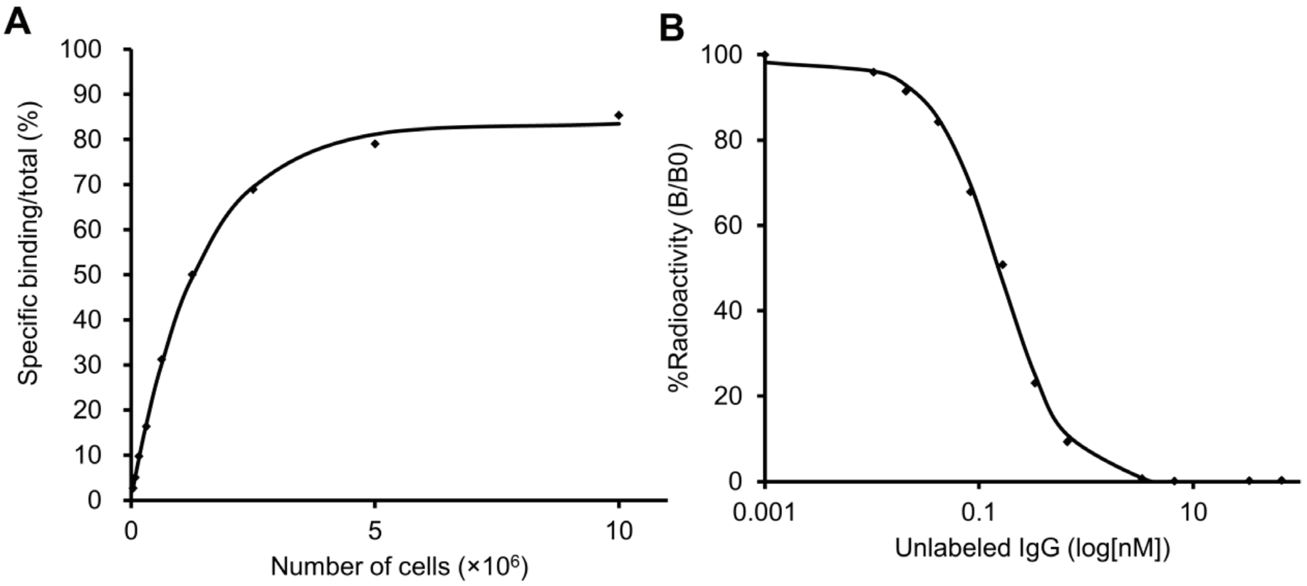

Supplementary Figure 1: *In vitro* characterization of ^{64}Cu-PCTA-cetuximab binding with HCT116-RFP cells. (**A**) Cell-binding assay of ^{64}Cu-PCTA-cetuximab with HCT116-RFP cells. The immunoreactive fraction was 96.4%. (**B**) Competitive-inhibition assay of ^{64}Cu-PCTA-cetuximab with HCT116-RFP cells. The dissociation constant was 0.097 nM.

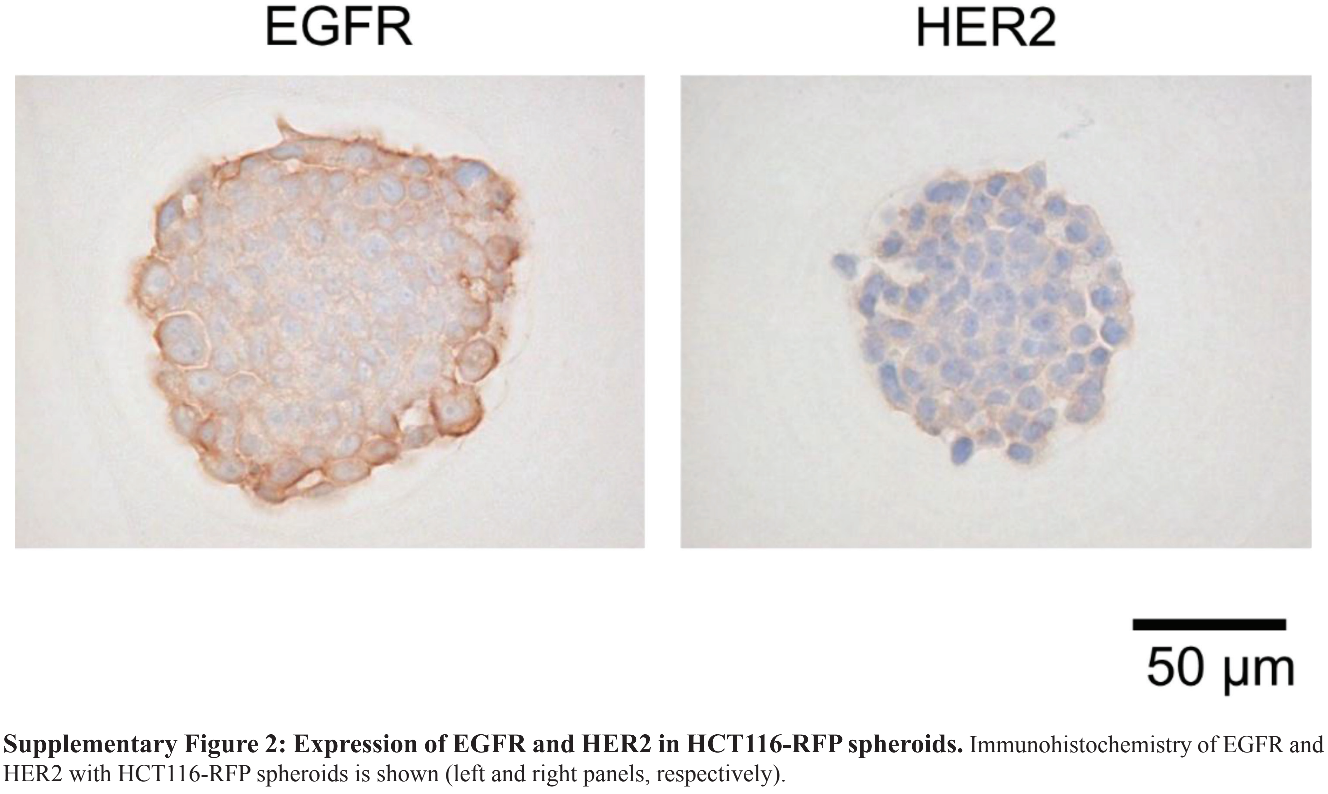

Supplementary Figure 2: Expression of EGFR and HER2 in HCT116-RFP spheroids. Immunohistochemistry of EGFR and HER2 with HCT116-RFP spheroids is shown (left and right panels, respectively).

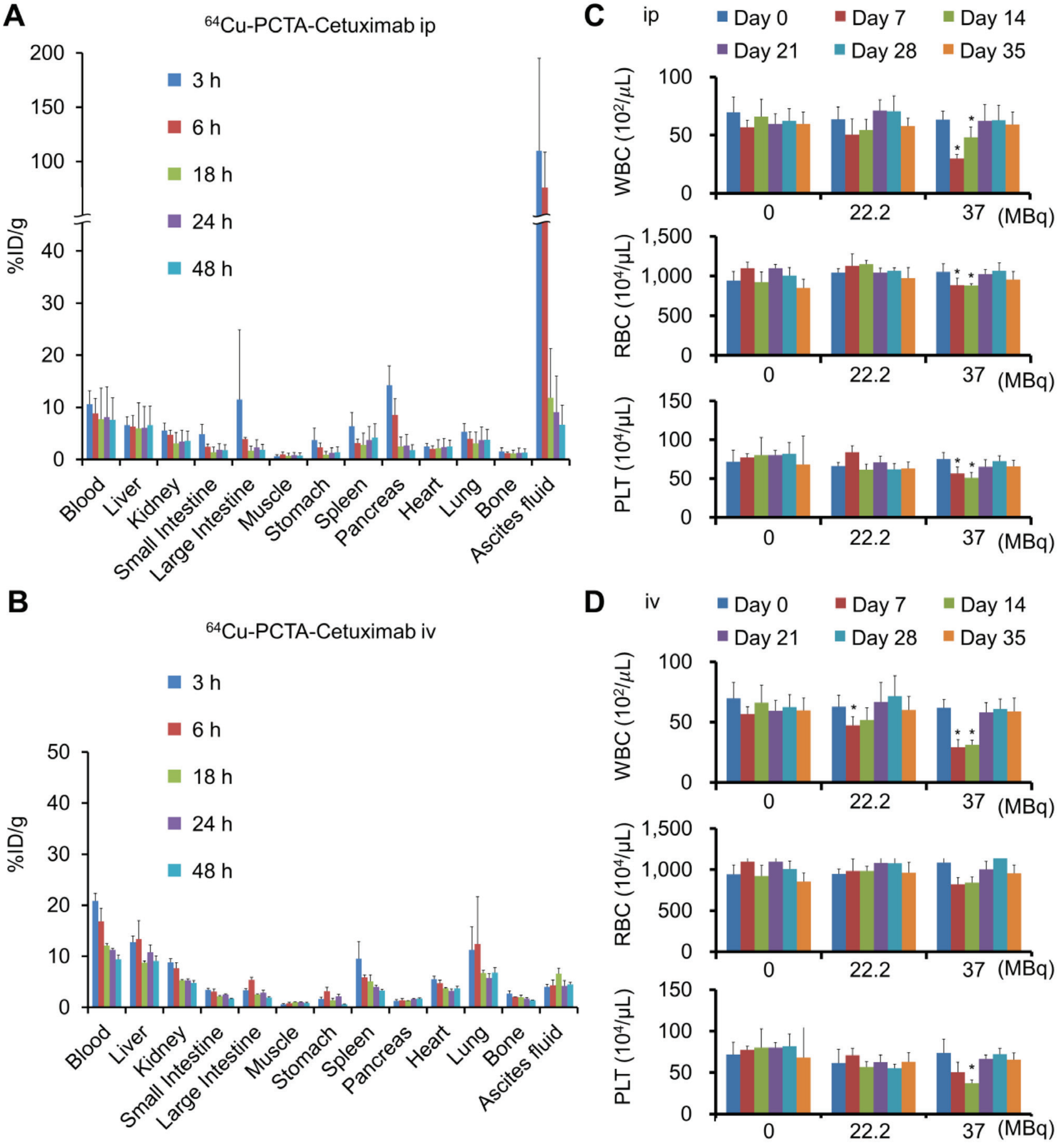

Supplementary Figure 3: Distribution and safety of ip-injected ^{64}Cu-PCTA-cetuximab. (**A**, **B**) Distribution of ^{64}Cu-PCTA-cetuximab in the organs of interest in mice following ip or iv administration at 3, 6, 18, 24, and 48 h ($n = 4$). Values are shown as the mean ± SD. (**C**) (**D**) Haematological toxicity in mice administered ^{64}Cu-PCTA-cetuximab by ip or iv injection. The numbers of WBCs, RBCs, and PLTs at day 0 (before ^{64}Cu-PCTA-cetuximab injection) and at 7, 14, 21, 28, and 35 days after ^{64}Cu-PCTA-cetuximab injection ($n = 4$) are shown. Values are shown as the mean ± SD. $^{*}P < 0.05$ vs day 0 in each group (1-way ANOVA with Bonferroni's post hoc test).

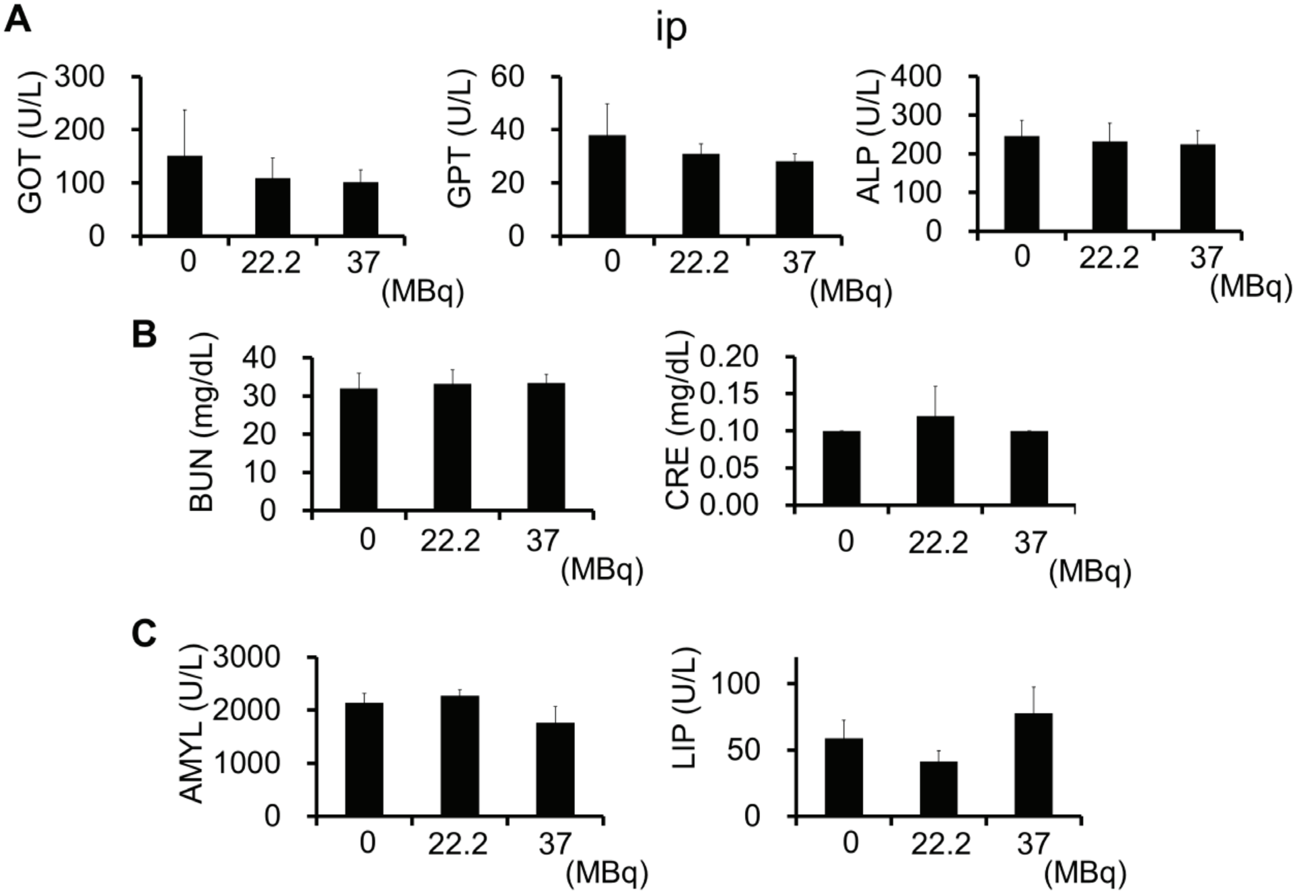

Supplementary Figure 4: Biochemical parameters in mice administered ^{64}Cu-PCTA-cetuximab by ip injection, measured at day 35. (**A**) Levels of GOT, GPT, and ALP as a measure of liver functions. (**B**) Levels of BUN and CRE for kidney functions. (**C**) Levels of AMYL and LIP for pancreas functions.

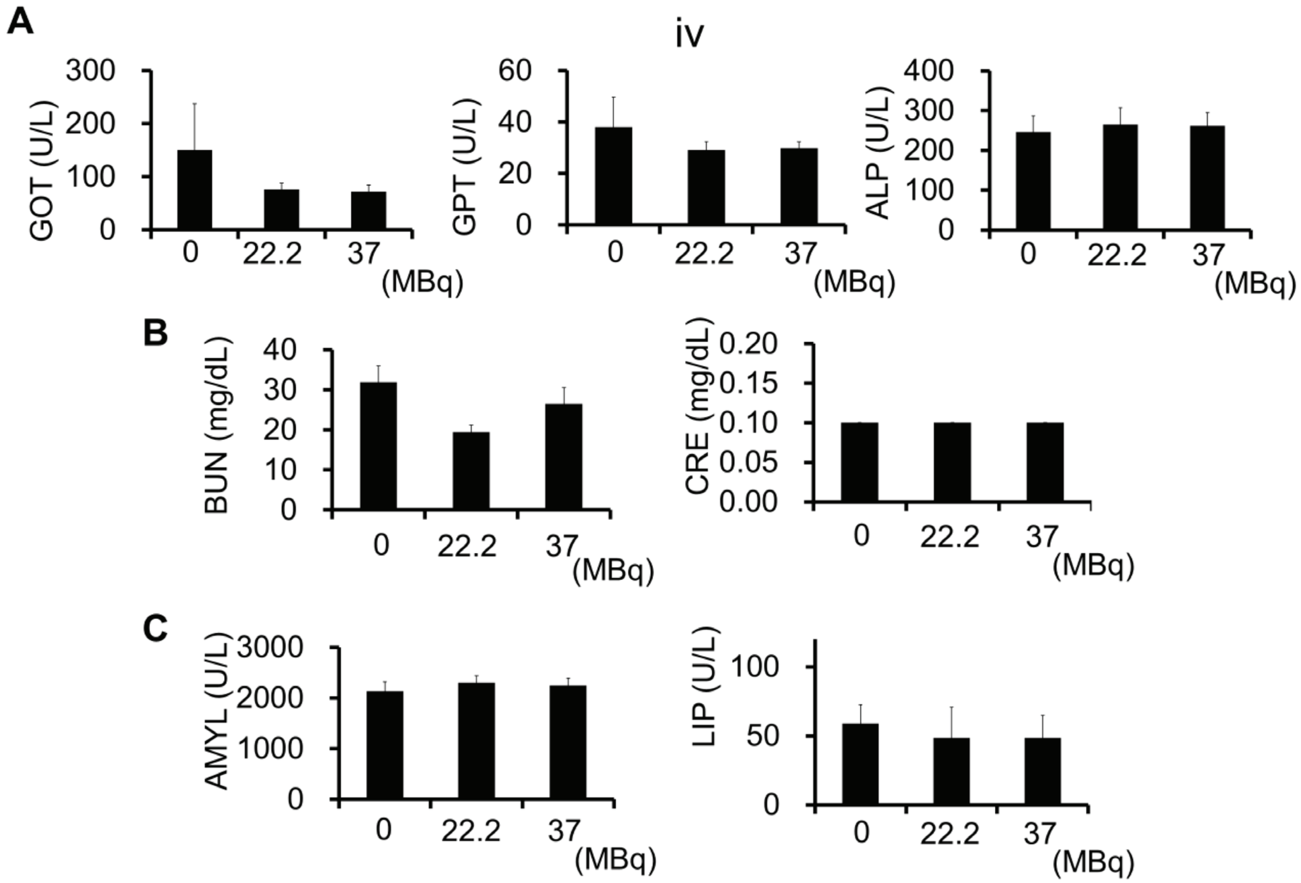

Supplementary Figure 5: Biochemical parameters in mice administered ^{64}Cu-PCTA-cetuximab by iv infection, measured at day 35. (**A**) Levels of GOT, GPT, and ALP as a measure of liver functions. (**B**) Levels of BUN and CRE for kidney functions. (**C**) Levels of AMYL and LIP for pancreas functions.

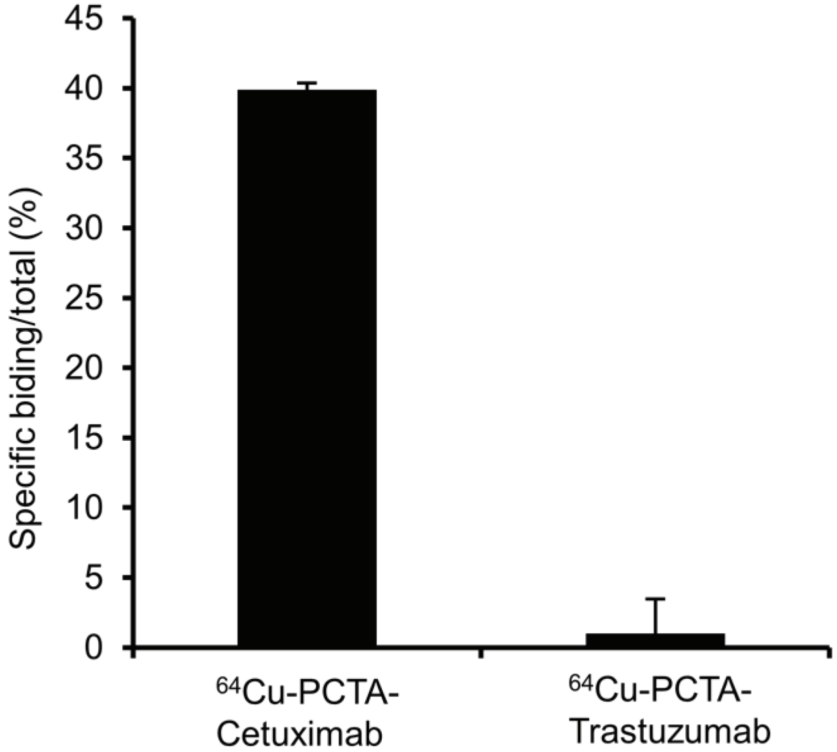

Supplementary Figure 6: Cell-binding affinity of ^{64}Cu-PCTA-cetuximab and ^{64}Cu-PCTA-trastuzumab for HCT116-RFP cells.

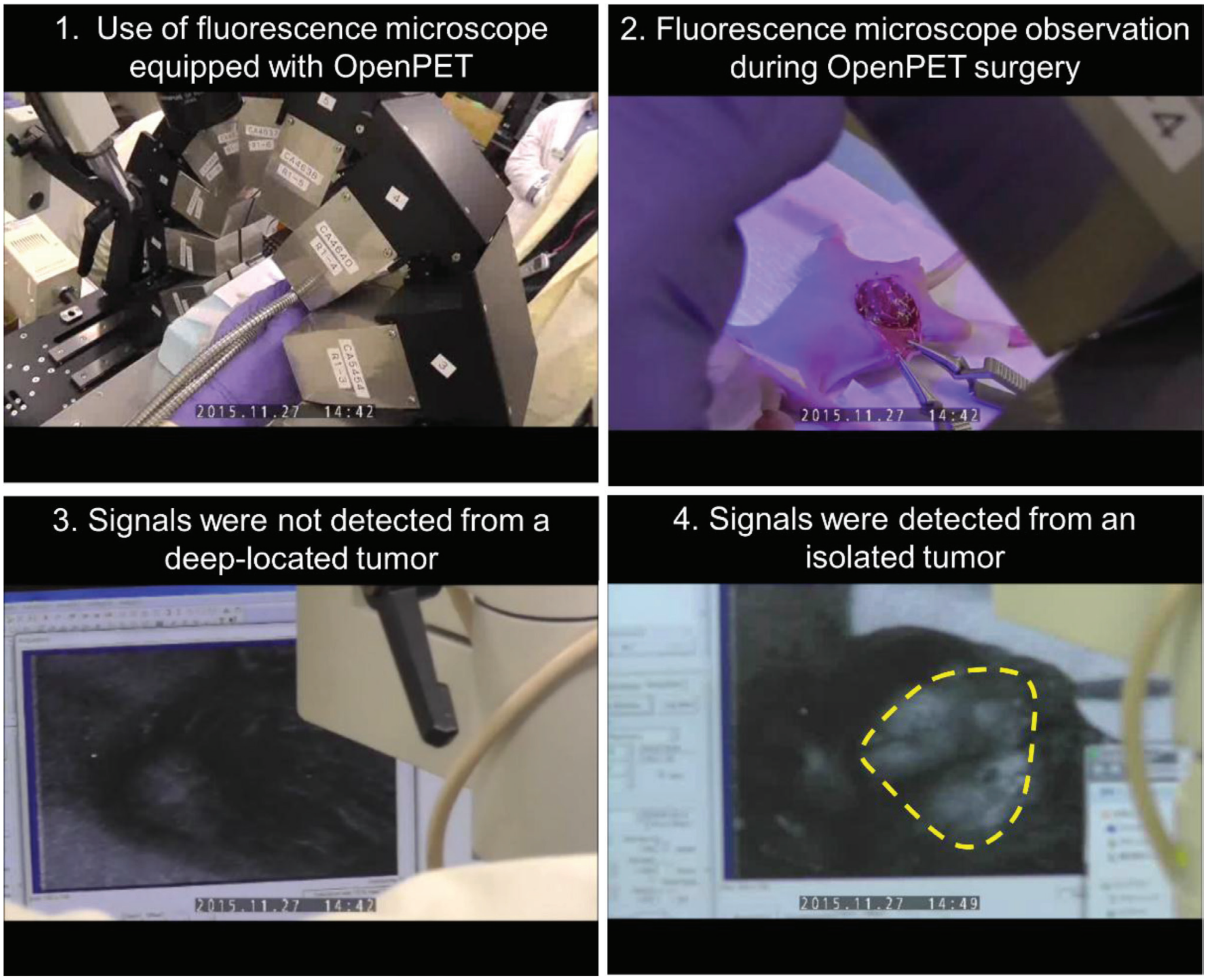

Supplementary Figure 7: Fluorescence imaging during OpenPET surgery. The images show a series of observations obtained with a fluorescence microscope equipped with the OpenPET system (upper; left and right panels), fluorescent imaging of tumours deeply located (lower, left), and fluorescent imaging of tumours identified during OpenPET surgery (lower, right). Fluorescent signals were detected from isolated tumours, but not deeply located tumours. The yellow, dotted circle highlights an isolated tumour.

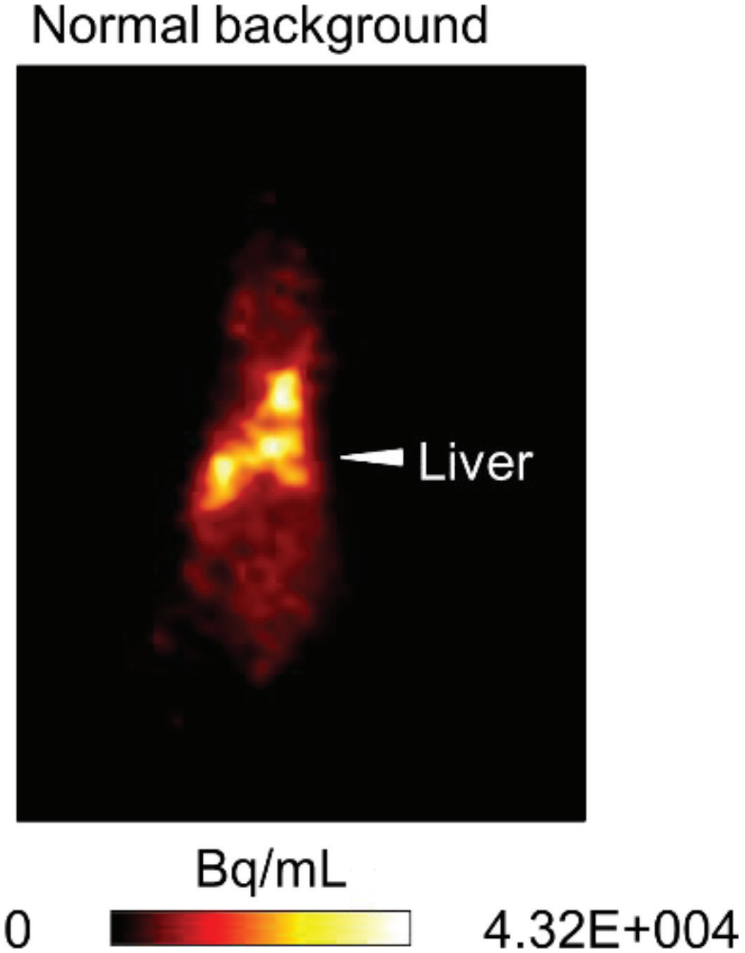

Supplementary Figure 8: OpenPET imaging with a tumour-free mouse administered ^{64}Cu-PCTA-cetuximab. An imaging dose of ^{64}Cu-PCTA-cetuximab (7.4 MBq/mouse) was administered to a mouse at 24 h before OpenPET imaging in a manner similar to that used in the OpenPET experiments represented in Figures 3, 4.
